# 

**DOI:** 10.1192/bjb.2020.84

**Published:** 2020-12

**Authors:** Erin Gourley

**Affiliations:** Core Trainee Year 1 (CT1) in psychiatry with Coventry and Warwickshire Partnership Trust, Coventry, UK. Email: e.gourley@nhs.net

Award-winning investigative journalist Robert Kolker follows the success of *Lost Girls* with the incredible story of the Galvin family and the impact of schizophrenia on their lives.

Kolker follows the story of Don and Mimi Galvin who, along with their 12 children, represented the ‘perfect all-American family’ – that is until 6 of their sons developed schizophrenia. After a whistle-stop tour of the history of psychology and psychiatry, Kolker deftly navigates us from the first Galvin brother's diagnosis in the 1960s through many pharmacological, genetic and psychological advances right up to our present-day understanding of schizophrenia. The book examines the conflicts within psychiatry over the years, poignantly portraying a family lost in the gulf of a specialty at war with itself.

Kolker deals sensitively and compassionately with the more challenging themes of sexual abuse, suicide and even murder, presenting the differing experiences of many of the family members in a thoughtful and considered way. A real strength of this book is that it also considers the experiences of ‘well’ family members, whose lives are so affected by their brothers’ illness. The hero of this story is undoubtedly Mimi Galvin: dismissed early on as the typical ‘schizophrenogenic mother’, she provides a lesson in love, loss and resilience and reminds us of the lengths to which a mother will go to protect her family.

Despite being marketed to the general public, the skilful mix of scientific detail coupled with engaging storytelling makes this book essential reading, particularly for psychiatry trainees. In addition to its clear educational value, for me this book served as a reminder of the importance of compassion in our services – not only to the patients themselves but to all those involved in their care.

You need look no further than the acknowledgements section to see the time, effort and genuine care that went into putting this story together with the help of the remaining Galvin family members. I believe this is something that, as psychiatrists, we can also bring to assembling and understanding our own patients’ stories.

